# Baltimore College of Dental Surgery

**Published:** 1882-05

**Authors:** 


### PROCEEDINGS.

### BALTIMORE COLLEGE OF DENTAL SURGERY.

The forty-second annual commencement.

The number of matriculates for the session was ninety-three.

The degree of D.D.S. was conferred on the following members of the class by Professor F. J. S. Gorgas, Dean of the Faculty:

| NAME. | RESIDENCE. | NAME. | RESIDENCE. |
|---|---|---|---|
| Henry S. Abendschein | Maryland. | Archie McAlpine | Pennsylvania. |
| Charles L. Alexander | North Carolina. | James P. McDonald | Mississippi. |
| Pedro A. Arcentales | South Carolina. | John Miller | New York. |
| Julius A. Ballentine | North Carolina. | George E. Morrow | Maryland. |
| John S. Bizzell | North Carolina. | Steuart B. Muncaster | Dist. Columbia. |
| Gordon H. Claude | Maryland. | Gustavus North | Iowa. |
| W. Connor Cleckley | South Carolina. | George A. Patrick | Georgia. |
| Genaro W. Cook | South America. | Hugh Pirkey | Virginia. |
| Charles W. Daly | B. West Indies. | W. Chalmers Ralston | Pennsylvania. |
| Amos C. Daniels | Pennsylvania. | B. Taylor Read | New York. |
| Willie F. Davison | Virginia. | Norman J. Roberts | Illinois. |
| William H. DeFord | Dist. Columbia. | Jose J. Sanjurjo | West Indies. |
| Charles F. Dinger | Maryland. | Samuel P. Sharp | Tennessee. |
| Louis P. Dotterer | South Carolina. | James E. Shields | North Carolina. |
| Thomas S. Eader | Maryland. | Charles A. Slocum | New York. |
| Wallace W. Freeman | Maryland. | Henderson Snell | North Carolina. |
| Ferdinand S. Gorgas | Pennsylvania. | Mordecai G, Sykes | Maryland. |
| G. Ashman Hamill | West Virginia. | George G. Taylor | Virginia. |
| Irby Hardy | Virginia. | Lewellen C. Tucker | Virginia |
| Lewis J. Harmanson | Virginia. | T, John Welch | Virginia. |
| William Hepburn, Jr. | New York. | B. H. Whittington | Maryland. |
| George W. Hunt | Pennsylvania. | Robt. C. Williams, M.D. | South Carolina. |
| Philip F. Laugenour | North Carolina. | John M. Wilson | Pennsylvania. |
| Cincero R. Yearick | Ohio. | | |

